# Performance Evaluation of Adaptive Tracking Techniques with Direct-State Kalman Filter †

**DOI:** 10.3390/s22020420

**Published:** 2022-01-06

**Authors:** Iñigo Cortés, Johannes Rossouw van der Merwe, Elena Simona Lohan, Jari Nurmi, Wolfgang Felber

**Affiliations:** 1 Satellite Based Positioning Systems Department, Fraunhofer IIS, Nordostpark 84, 90411 Nuremberg, Germany; Johannes.roussouw.vandermerwe@iis.fraunhofer.de (J.R.v.d.M.); wolfgang.felber@iis.fraunhofer.de (W.F.); 2 Electrical Engineering, Tampere University, 33014 Tampere, Finland; elena-simona.lohan@tuni.fi (E.S.L.); jari.nurmi@tuni.fi (J.N.)

**Keywords:** global navigation satellite system (GNSS), scalar tracking loop (STL), direct-state Kalman filter (DSKF), lookup table direct-state Kalman filter (LUT-DSKF), loop-bandwidth control algorithm (LBCA), adaptive tracking techniques

## Abstract

This paper evaluates the performance of robust adaptive tracking techniques with the direct-state Kalman filter (DSKF) used in modern digital global navigation satellite system (GNSS) receivers. Under the assumption of a well-known Gaussian distributed model of the states and the measurements, the DSKF adapts its coefficients optimally to achieve the minimum mean square error (MMSE). In time-varying scenarios, the measurements’ distribution changes over time due to noise, signal dynamics, multipath, and non-line-of-sight effects. These kinds of scenarios make difficult the search for a suitable measurement and process noise model, leading to a sub-optimal solution of the DSKF. The loop-bandwidth control algorithm (LBCA) can adapt the DSKF according to the time-varying scenario and improve its performance significantly. This study introduces two methods to adapt the DSKF using the LBCA: The LBCA-based DSKF and the LBCA-based lookup table (LUT)-DSKF. The former method adapts the steady-state process noise variance based on the LBCA’s loop bandwidth update. In contrast, the latter directly relates the loop bandwidth with the steady-state Kalman gains. The presented techniques are compared with the well-known state-of-the-art carrier-to-noise density ratio ($C/N_{0}$)-based DSKF. These adaptive tracking techniques are implemented in an open software interface GNSS hardware receiver. For each implementation, the receiver’s tracking performance and the system performance are evaluated in simulated scenarios with different dynamics and noise cases. Results confirm that the LBCA can be successfully applied to adapt the DSKF. The LBCA-based LUT-DSKF exhibits superior static and dynamic system performance compared to other adaptive tracking techniques using the DSKF while achieving the lowest complexity.

## 1. Introduction

Global navigation satellite system (GNSS) receivers synchronize with GNSS signals to decode the navigation message, measure the pseudo-range and pseudo-range rate, and calculate a position, velocity, and time (PVT) solution [1,2]. The synchronization consists of two stages: Acquisition and tracking. Acquisition performs a coarse estimate of the synchronization parameters, whereas the tracking stage provides an improved estimate. The latter stage uses the scalar tracking loop (STL) to refine the synchronization of the incoming GNSS signals [3,4]. The STL replicates a synchronization parameter for every loop iteration. The synchronization lock is achieved when the difference between the true parameter and its replica (i.e., the estimation error) tends to zero [3]. The carrier phase θ, the carrier Doppler $f_{d}$, and the code phase τ are the GNSS signal parameters in which the GNSS receiver must synchronize. Therefore, a tracking channel comprises three STLs: phase locked loop (PLL), frequency locked loop (FLL), and delay locked loop (DLL). A correlator, a discriminator, a loop filter, and a numerically controlled oscillator (NCO) compose the STL [5,6]. The STL’s configuration parameters are the type of discriminator, the loop bandwidth *B*, the integration time $\tau_{\mathrm{int}}$, the order *p*, and the correlator spacing. These parameters determine the robustness against noise and signal dynamics. The well-known trade-off between noise filtering capabilities and signal dynamics resistance is the main problem of standard STLs with fixed configurations [7]. For instance, a high-order STL with wide loop bandwidth and short integration time is adequate to track rapidly changing parameters. In contrast, a low-order STL with narrow loop bandwidth and long integration time is preferable to track noisy parameters.

Time-varying scenarios are characterized by different realizations of signal dynamics, noise, and fading effects. These changing effects challenge the synchronization capability of the tracking stage [1,7]. Since traditional tracking lacks resilience due to its fixed configuration, there has been significant research towards robust tracking solutions to solve this problem [8,9]. However, there is still ample investigation to find the optimal technique in terms of performance and complexity [7].

The Kalman filter (KF) is an optimal infinite impulse response (IIR) estimator under the assumption of linear Gaussian error statistics [10,11,12]. Good knowledge of the process noise covariance Q and measurement noise covariance R allows the KF to optimally adapt its coefficients to achieve the minimum mean square error (MMSE) [13]. There are several KF implementation methods in STLs [14] grouped into error-state Kalman-filter (ESKF) and direct-state Kalman filter (DSKF) [15]. The former replaces the loop filter of the STL with a KF whereas the latter considers the whole STL part of the KF. In the ESKF, the measurement is the discriminator’s output, and the predicted measurement drives the NCO. A detailed study of this architecture has been done in previous research [16,17,18,19]. The complexity of the ESKF is a limiting factor but it can be reduced taking advantage of the Kalman gains’ convergence in the steady-state [19]. The DSKF is more straightforward to implement than the ESKF since it considers the whole STL part of the KF. In this case, the measurement is the synchronization parameter, the innovation is the discriminator’s output, and the loop filter and the NCO are the core of the KF. This method benefits from the simplicity of relating the coefficients of the standard STL with the Kalman gains of the DSKF [20].

The MMSE is only achieved if á priori knowledge of Q and R is available or if these are accurately estimated [13]. If this is not the case, the KF tends to be a sub-optimal solution [21]. The difficulty of finding the correct Q and R values increases even more in time-varying scenarios since these parameters are continuously changing [20]. Different methods to estimate the noise covariances of the KF have been summarized in a review study [22]. One solution can be to implement a moving average filter to estimate Q and R [23]. Moreover, it is possible to implement a carrier-to-noise density ratio (*C*/*N*_0_)-based DSKF, in which R depends on the variance of the STL discriminator’s output [24]. Q can also be adapted according to the dynamic stress error [25]. Another solution is the implementation of an ESKF combining long non-coherent integrations to improve tracking sensitivity [26]. Moreover, a weighted adaptive ESKF can be implemented for scenarios with unknown *C*/*N*_0_ [27].

**Figure 1:**
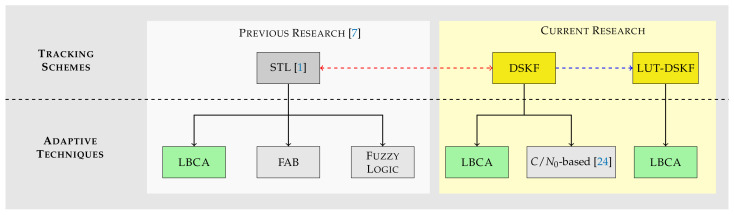
Research roadmap and comparison to other publications.

Figure 1 presents a roadmap of the studies. Previous research [7] evaluated three adaptive tracking techniques in the standard STL: the loop-bandwidth control algorithm (LBCA), the fast adaptive bandwidth (FAB), and the Fuzzy logic. The LBCA was superior to the other techniques regarding tracking performance, system performance, and complexity. This technique adjusts the STL’s loop bandwidth based on the statistics of the discriminator’s output [28]. The current research extends the LBCA by implementing it in the DSKF. The relationship between the STL and the DSKF, referred to as the dashed red line, facilitates the LBCA’s implementation in the DSKF. The LBCA’s estimated loop bandwidth can be related to Q and R in the steady-state. Suppose one of the covariances is set to a constant value. In that case, the remainder covariance can be updated based on the LBCA’s bandwidth update.

In addition, the DSKF can be simplified considering the convergence of the Kalman gains in the steady-state, referred to as the dashed blue line. This low-complexity tracking scheme is the so-called lookup table (LUT)-DSKF. The LBCA can also be implemented in the LUT-DSKF and directly update the Kalman gains based on the estimated loop bandwidth. This paper evaluates the performance of the LBCA-based DSKF and the LBCA-based LUT-DSKF. These adaptive techniques are compared with the *C*/*N*_0_-based DSKF, a well-known state-of-the-art method that adapts R based on the estimated *C*/*N*_0_ [24]. The DSKF, the LUT-DSKF, and the mentioned adaptive techniques are implemented in the carrier phase synchronization tracking stage of the GOOSE© receiver [29]. Each adaptive technique’s tracking performance and system performance are evaluated under simulated scenarios with different dynamics and noise levels. These methods are also compared with the LBCA-based standard STL [7].

This research expands a conference paper [20] by describing in detail the relationship between STL and the DSKF, explaining the DSKF’s steady-state convergence that leads to the LUT-DSKF, adapting the LUT-DSKF using the LBCA, and improving the scope of the results.

The rest of the paper is organized as follows: Section 2 compares the standard STL with the DSKF, analyzes the steady-state convergence and derives the tracking scheme of the LUT-DSKF. Section 3 shows the architecture of the adaptive techniques used in the DSKF and the LUT-DSKF. The experimental setup and implementation in an open software interface GNSS hardware receiver are described in Section 4. Section 5 presents the adaptive tracking techniques’ complexity, tracking performance, and system performance results. These results are discussed in Section 6. Finally, Section 7 concludes and indicates future work.

## 2. Tracking Scheme of Direct-State Kalman Filter

The DSKF considers the standard STL as part of the KF [15]. This section describes the relation between standard STL and DSKF and analyzes the DSKF’s convergence in the steady-state. First, an overview of the STL’s open-loop state space model (SSM) and transfer function will facilitate the comparison of STL with the DSKF. Second, the DSKF is explained, and the equivalence to a standard STL is proven. Third, the process noise covariance matrix Q and measurement noise covariance matrix R are described. Fourth, the Kalman filter’s discrete algebraic Riccati equation (DARE) solution leads to the relation between the bandwidth *B*, the Kalman gains K, R, and Q. Finally, from the DARE’s solution, the LUT-DSKF is presented.

### 2.1. Standard Scalar Tracking Loop

**Figure 2:**
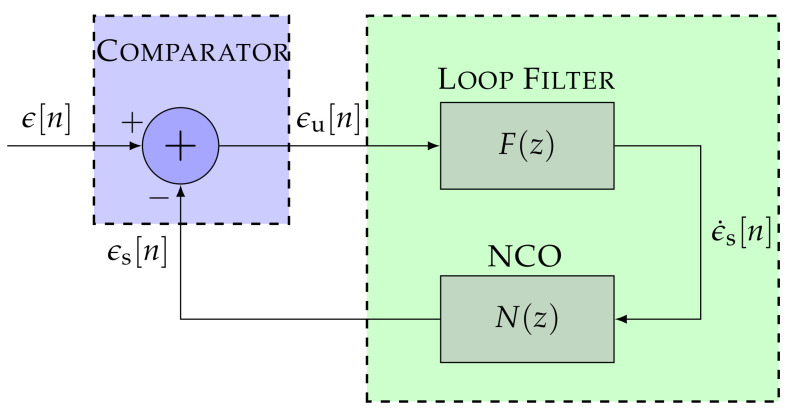
Linear model of the scalar tracking loop (STL).

The STL’s tracking scheme must be revisited to understand the relationship between the standard STL and the DSKF. Figure 2 shows the block diagram of the STL’s linear model. The comparator is a linearized discriminator that performs the difference between the input signal’s synchronization parameter ϵ and the estimated smoothed error $\epsilon_{s}$:

(1) $$\begin{array}{c} \epsilon_{u}\left[n\right]=\epsilon \left[n\right]-\epsilon_{s}\left[n\right] \end{array}$$

where *n* is the sample index, and $\epsilon_{u}$ is the un-smoothed error that outputs the comparator. The loop filter smooths $\epsilon_{u}$ and drives the smoothed error rate ${\dot{\epsilon }}_{s}$ to the NCO. Finally, the NCO closes the loop, sending $\epsilon_{s}$ to the comparator. Depending on the type of discriminator, ϵ represents the carrier offset (PLL), the frequency Doppler (FLL), or the code phase offset (DLL).

Considering that the backward Euler transform (BET) is used to discretize the STL [30], the open-loop digital SSM representation of a $p^{th}$-order STL is expressed as follows:

(2) x1[n]x2[n]⋮xp[n]1τintτint2⋯τintp−101τint⋯τintp−2⋮⋮⋱⋱⋮000⋯τint000⋯1︸x[n]=1τintτint2⋯τintp−101τint⋯τintp−2⋮⋮⋱⋱⋮000⋯τint000⋯1︸Ax1[n−1]x2[n−1]⋮xp[n−1]1τintτint2⋯τintp−101τint⋯τintp−2⋮⋮⋱⋱⋮000⋯τint000⋯1︸x[n−1]+αp−1αp−2⋮α01τintτint2⋯τintp−101τint⋯τintp−2⋮⋮⋱⋱⋮000⋯τint000⋯1︸ατintϵu[n]

(3) $$\begin{array}{c} \epsilon_{s}\left[n\right]=\underset{H}{\underset{︸}{\left[\begin{array}{cccc} 1 & 0 & \cdots & 0 \end{array}\right]}}\;A\;x\left[n-1\right] \end{array}$$

(4) $$\begin{array}{c} x\in {ℜ}^{p\times 1}\;H\in {ℜ}^{1\times p}\;A\in {ℜ}^{p\times p} \end{array}$$

where x is the state vector, A is the state transition matrix, α is the filter coefficients vector, and H is the observation matrix.

To calculate the STL’s open-loop transfer function $H_{o}\left(z\right)$, the z-transform Z(·) of Equations (2) and (3) must be performed first:

(5) $$Z\left(x\right)={\left(I-A\;z^{-1}\right)}^{-1}\;\alpha \;\tau_{\mathrm{int}}\;Z\left(\epsilon_{u}\right)$$

(6) $$Z\left(\epsilon_{s}\right)=H\;A\;z^{-1}\;Z\left(x\right)$$

Second, Equations (5) and (6) are combined:

(7) $$Z\left(\epsilon_{s}\right)=H\;A\;{\left(I-A\;z^{-1}\right)}^{-1}\;\alpha \;\tau_{\mathrm{int}}\;z^{-1}\;Z\left(\epsilon_{u}\right)$$

Finally, the open-loop transfer function $H_{o}\left(z\right)$ is expressed as:

(8) $$\begin{array}{cc} H_{o}\left(z\right) & =Z\left(\frac{\epsilon_{s}}{\epsilon_{u}}\right)=H\;A\;{\left(I-A\;z^{-1}\right)}^{-1}\;\alpha \;\tau_{\mathrm{int}}\;z^{-1} \end{array}$$

(9) $$\begin{array}{cc} \; & =\sum_{l=0}^{p-1}\frac{\alpha_{l}\tau_{\mathrm{int}}^{p-l}z^{-1}}{{\left(1-z^{-1}\right)}^{p-l}} \end{array}$$

(10) =∑l=0p−1αlτintp−l−1(1−z−1)p−l−1︸F(z)·τintz−11−z−1∑k=0p−1αkτintp−k−1(1−z−1)p−k−1︸N(z)

where F(z) and N(z) indicate the loop filter’s and NCO’s transfer function.

The closed-loop transfer function $H_{c}\left(z\right)$ is related to $H_{o}\left(z\right)$ considering the z-transform of Equation (1):

(11) $$\begin{array}{cc} H_{c}\left(z\right) & =Z\left(\frac{\epsilon }{\epsilon_{u}}\right)=\frac{H_{o}\left(z\right)}{1+H_{o}\left(z\right)} \end{array}$$

**Figure 3:**
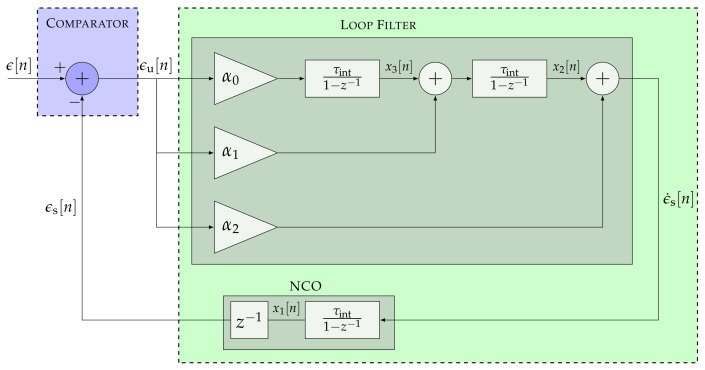
Linear model of a third-order STL.

Figure 3 presents a particular case of the STL with p=3.

The open-loop SSM of a third-order STL is derived from Equations (2) and (3):

(12) $$\begin{array}{c} \left[\begin{array}{c} x_{1}\left[n\right]\\ x_{2}\left[n\right]\\ x_{3}\left[n\right] \end{array}\right]=\left[\begin{array}{ccc} 1 & \tau_{\mathrm{int}} & \tau_{\mathrm{int}}^{2}\\ 0 & 1 & \tau_{\mathrm{int}}\\ 0 & 0 & 1 \end{array}\right]\left[\begin{array}{c} x_{1}\left[n-1\right]\\ x_{2}\left[n-1\right]\\ x_{3}\left[n-1\right] \end{array}\right]+\left[\begin{array}{c} \alpha_{2}\\ \alpha_{1}\\ \alpha_{0} \end{array}\right]\;\tau_{\mathrm{int}}\;\epsilon_{u}\left[n\right] \end{array}$$

(13) $$\begin{array}{c} \epsilon_{s}\left[n\right]=\left[\begin{array}{ccc} 1 & \tau_{\mathrm{int}} & \tau_{\mathrm{int}}^{2} \end{array}\right]\;x\left[n-1\right] \end{array}$$

Suppose the third-order STL is used for carrier phase synchronization. In that case, $x_{1}$, $x_{2}$, and $x_{3}$ represent the carrier phase, carrier Doppler, and carrier Doppler rate.

Moreover, based on Equation (10), the open-loop transfer function of the third-order STL $H_{o_{3}}$ is expressed as:

(14) $$\begin{array}{c} H_{o_{3}}\left(z\right)=\frac{\alpha_{2}\tau_{\mathrm{int}}{\left(1-z^{-1}\right)}^{2}+\alpha_{1}\tau_{\mathrm{int}}^{2}\left(1-z^{-1}\right)+\alpha_{0}\tau_{\mathrm{int}}^{3}}{{\left(1-z^{-1}\right)}^{3}} \end{array}$$

Consequently, the closed-loop transfer function $H_{c_{3}}$ is calculated based on Equation (11):

(15) $$\begin{array}{c} H_{c_{3}}\left(z\right)=\frac{\alpha_{2}\tau_{\mathrm{int}}{\left(1-z^{-1}\right)}^{2}+\alpha_{1}\tau_{\mathrm{int}}^{2}\left(1-z^{-1}\right)+\alpha_{0}\tau_{\mathrm{int}}^{3}}{{\left(1-z^{-1}\right)}^{3}+\alpha_{2}\tau_{\mathrm{int}}{\left(1-z^{-1}\right)}^{2}+\alpha_{1}\tau_{\mathrm{int}}^{2}\left(1-z^{-1}\right)+\alpha_{0}\tau_{\mathrm{int}}^{3}} \end{array}$$

### 2.2. Direct-State Kalman Filter

The DSKF, as in the KF [10,11,12], is divided into two stages: prediction and update. The prediction step estimates the predicted state $\hat{x}\left[n\right]$ and predicted error covariance $\hat{P}\left[n\right]$. $\hat{x}\left[n\right]$ and $\hat{P}\left[n\right]$ are calculated based on the previously updated state x[n−1], the previously updated error covariance P[n−1], and the process noise covariance matrix Q[n]:

(16) $$\hat{x}\left[n\right]=A\;x\left[n-1\right]$$

(17) $$\hat{P}\left[n\right]=A\;P\left[n-1\right]\;A^{T}+Q\left[n\right]$$

(18) $$\begin{array}{c} \hat{x}\in {ℜ}^{p\times 1}\;\left\{\hat{P},\;P,\;Q\right\}\in {ℜ}^{p\times p} \end{array}$$

where the superscript ${\left(\cdot \right)}^{T}$ is the transpose.

The update stage calculates the updated state x[n] based on $\hat{x}\left[n\right]$, the measurement residual $\epsilon_{u}\left[n\right]$, and the Kalman gains K[n]. $\epsilon_{u}\left[n\right]$ comes from the difference between the observations ϵ[n] and the estimated measurement based on $\hat{x}\left[n\right]$. The Kalman gains K[n] indicate how accurate the measurements are. K[n] depends on the predicted error covariance $\hat{P}\left[n\right]$ and the measurement noise covariance R[n].

(19) $$\epsilon_{u}\left[n\right]=\epsilon \left[n\right]-H\;\hat{x}\left[n\right]$$

(20) $$S\left[n\right]=H\;\hat{P}\left[n\right]\;H^{T}+R\left[n\right]$$

(21) $$K\left[n\right]=\hat{P}\left[n\right]\;H^{T}\;S^{-1}\left[n\right]$$

(22) $$x\left[n\right]=\hat{x}\left[n\right]+K\left[n\right]\;\epsilon_{u}\left[n\right]$$

(23) $$P\left[n\right]=\left(I-K\left[n\right]\;H\right)\;\hat{P}\left[n\right]$$

(24) $$\begin{array}{c} \left\{\epsilon_{u},\;\epsilon \right\}\in {ℜ}^{m\times 1}\;\left\{S,\;R\right\}\in {ℜ}^{m\times m}\;H\in {ℜ}^{m\times p}\;K\in {ℜ}^{p\times m}\;I\in {ℜ}^{p\times p} \end{array}$$

where S[n] is the innovation covariance matrix, and I is the identity matrix. The order *p* and the number of measurements *m* determine the dimension of the presented variables. This paper assumes only one measurement (i.e., m=1) to compare the DSKF with the STL. In that case, Equation (1) is the same as Equation (19).

**Figure 4:**
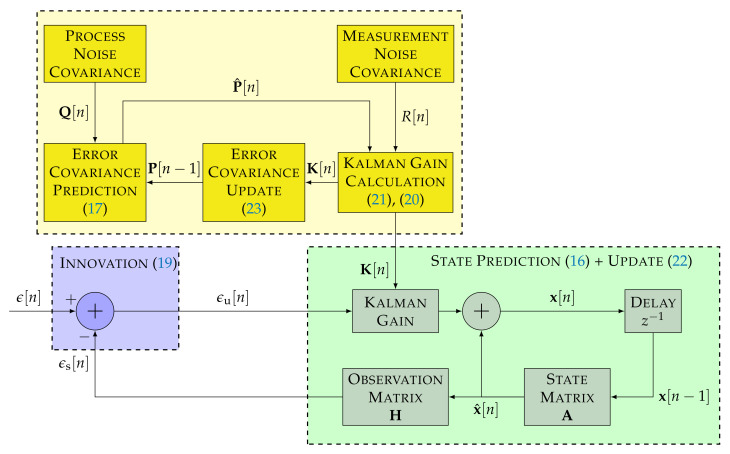
Linear model of the direct-state Kalman filter (DSKF) with m=1. ©IEEE. Adapted, with permission, from [20].

Figure 4 shows the linear model of the DSKF considering only one measurement. The innovation block of the DSKF is equivalent to the STL’s comparator of Figure 2. The state prediction and update block marked in green correspond to the STL’s loop filter and NCO. The main difference between the standard STL and the DSKF is the addition of the Kalman gains’ calculation depicted in the dashed yellow block of Figure 4.

The DSKF’s open-loop SSM representation are obtained combining Equations (16) and (22):

(25) $$x\left[n\right]=A\;x\left[n-1\right]+K\left[n\right]\;\epsilon_{u}\left[n\right]$$

(26) $$\epsilon_{s}\left[n\right]=H\;\hat{x}\left[n\right]=H\;A\;x\left[n-1\right]$$

The SSM of the DSKF is equivalent to the STL’s SSM presented in Equations (2) and (3). From this equivalence, the Kalman gains K can be related to the STL’s filter coefficients α as:

(27) $$K=\alpha \;\tau_{\mathrm{int}}$$

**Figure 5:**
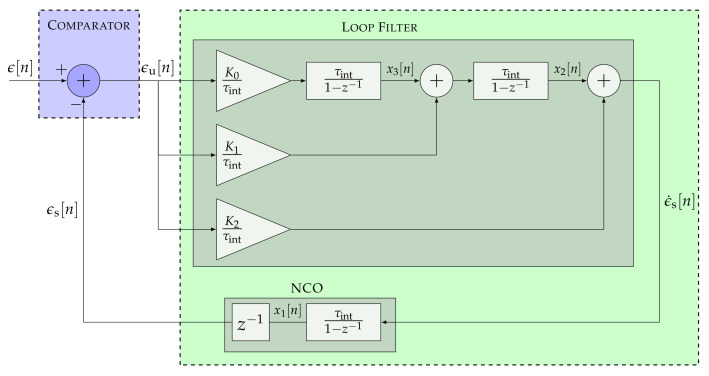
Linear model equivalence between a third-order DSKF and a third-order STL. ©IEEE. Adapted, with permission, from [20].

Figure 5 shows the linear model of a third-order DSKF (i.e., p=3). The open-loop SSM, the open-loop transfer function $H_{o_{3}}$, and the closed-loop transfer function $H_{c_{3}}$ are the same as Equations (12)–(15) due to the relation presented in Equation (27).

### 2.3. Process and Measurement Noise Covariance Matrix

The Kalman gains K are calculated for each loop iteration based on Equations (17), (20), (21) and (23). K depends on the process noise covariance Q and measurement noise covariance R. This paper calculates these matrices considering a third-order DSKF for carrier phase tracking. In addition, a constant-acceleration model is assumed [31,32] for the Q calculation. The BET is used to discretize the noise that is added in the acceleration state:

(28) $$w_{k}=A\;w\tau_{\mathrm{int}}=A\;\left[\begin{array}{c} 0\\ 0\\ w_{a} \end{array}\right]\;\tau_{\mathrm{int}}=\left[\begin{array}{c} w_{a}\tau_{\mathrm{int}}^{3}\\ w_{a}\tau_{\mathrm{int}}^{2}\\ w_{a}\tau_{\mathrm{int}} \end{array}\right]$$

where w is the continuous noise vector, $w_{k}$ is the discretized noise vector, and $w_{a}$ is the zero-mean Gaussian distributed perturbation that suffers the acceleration in $\mathrm{cycles}/s^{3}$.

Q is obtained performing the variance of the discretized noise:

(29) $$Q=E\left(A\;w\;w^{T}\;A^{T}\right)\tau_{\mathrm{int}}^{2}=\left[\begin{array}{ccc} \tau_{\mathrm{int}}^{6} & \tau_{\mathrm{int}}^{5} & \tau_{\mathrm{int}}^{4}\\ \tau_{\mathrm{int}}^{5} & \tau_{\mathrm{int}}^{4} & \tau_{\mathrm{int}}^{3}\\ \tau_{\mathrm{int}}^{4} & \tau_{\mathrm{int}}^{3} & \tau_{\mathrm{int}}^{2} \end{array}\right]\;q$$

where *q* is the variance of $w_{a}$ in ${\mathrm{cycles}}^{2}/s^{6}$ and determines the uncertainty of the states. A higher *q* implies higher state uncertainty, leading to higher confidence in the incoming measurements. On the contrary, a lower the *q* leads to higher confidence of the states and less dependence on the measurement.

The measurement noise covariance R determines the validity of the incoming measurements. A high value indicates an increased uncertainty, whereas a low value defines high confidence. An adequate model for R is the Cramér-Rao bound (CRB) of the STL since it represents the minimum error variance of a time of arrival (ToA) unbiased estimator [33,34]. In this case, the measurement residual of the DSKF is the discriminator’s output $\epsilon_{u}$ of an STL. The CRB of $\epsilon_{u}$ is achieved when the error estimation does not feedback on additional noise. Only the thermal noise of the incoming error parameter ϵ is present. If the carrier phase offset parameter (ϵ=θ) is taken as a measurement, and under the assumption of a two-quadrant discriminator, *R* in ${\mathrm{cycles}}^{2}$ is represented as [28,34,35]:

(30) $$\begin{array}{c} R=\mathrm{VAR}\left(\theta \right)=\frac{1}{2\tau_{\mathrm{int}}{\left(C/N_{0}\right)}_{l}}\left(1+\frac{1}{2\tau_{\mathrm{int}}{\left(C/N_{0}\right)}_{l}}\right) \end{array}$$

where ${\left(C/N_{0}\right)}_{l}$ is the linear *C*/*N*_0_ in Hertz. This relation is commonly used in *C*/*N*_0_-based DSKF [24,25].

### 2.4. Steady-State Analysis

In the steady-state region, the Kalman gains K converge to a steady-state value given a constant *q* and *R*. The solution of the DARE presents the relation between K, *q* and *R*. The expression of the DARE is [36,37]:

(31) $$\begin{array}{c} P_{\mathrm{ss}}=A\;P_{\mathrm{ss}}\;A^{T}-A\;P_{\mathrm{ss}}\;H^{T}{\left(H\;P_{\mathrm{ss}}\;H^{T}+R\right)}^{-1}H\;P_{\mathrm{ss}}\;A^{T}+Q \end{array}$$

where $P_{\mathrm{ss}}$ is the steady-state convergence of the error covariance matrix P. The following is assumed to facilitate DARE’s solution [19]:

(32) $$\begin{array}{c} R\gg H\;P_{\mathrm{ss}}\;H^{T} \end{array}$$

The resulting $P_{\mathrm{ss}}$ for a third-order DSKF is symmetric and defined as:

(33) $$\begin{array}{c} P_{\mathrm{ss}}=\left[\begin{array}{ccc} 2q^{1/6}R^{5/6}\tau_{\mathrm{int}} & 2q^{1/3}R^{2/3}\tau_{\mathrm{int}} & q^{1/2}R^{1/2}\tau_{\mathrm{int}}\\ \mathrm{sym}. & 3q^{1/2}R^{1/2}\tau_{\mathrm{int}} & 2q^{2/3}R^{1/3}\tau_{\mathrm{int}}\\ \mathrm{sym}. & \mathrm{sym}. & 2q^{5/6}R^{1/6}\tau_{\mathrm{int}} \end{array}\right] \end{array}$$

Applying Equations (32) and (33) into Equation (21), the steady-state Kalman gains $K_{\mathrm{ss}}$ are represented as:

(34) $$\begin{array}{c} K_{\mathrm{ss}}=P_{\mathrm{ss}}\;H^{T}\;R^{-1}={\left[\begin{array}{ccc} 2{\left(q/R\right)}^{1/6}\tau_{\mathrm{int}} & 2{\left(q/R\right)}^{1/3}\tau_{\mathrm{int}} & {\left(q/R\right)}^{1/2}\tau_{\mathrm{int}} \end{array}\right]}^{T} \end{array}$$

### 2.5. Equivalent Noise Bandwidth

The digital one-sided equivalent noise bandwidth $B_{d}$ is defined as [38,39,40]:

(35) $$\begin{array}{c} 2B_{d}\tau_{\mathrm{int}}=\frac{1}{2\pi j}\oint_{|z|=1}H_{c}\left(z\right)H_{c}\left(z^{-1}\right)z^{-1}dz \end{array}$$

Assuming that the integration time $\tau_{\mathrm{int}}$ tends to zero, the digital loop bandwidth is equivalent to the analog loop bandwidth *B* [38,39,40]. The relation between *B* and the coefficients of a third-order STL is expressed as:

(36) $$\begin{array}{c} \lim_{\tau_{\mathrm{int}}\to 0}B_{d}=B=\frac{\alpha_{2}^{2}\alpha_{1}-\alpha_{2}\alpha_{0}+\alpha_{1}^{2}}{4(\alpha_{2}\alpha_{1}-\alpha_{0})} \end{array}$$

Substituting Equations (27) and (34) in Equation (36), the relation between the steady-state equivalent noise bandwidth of a third-order DSKF, *q*, and *R* is [5]:

(37) $$\begin{array}{c} B_{\mathrm{ss}}=\frac{5}{6}\;\sqrt[{6}]{\frac{q}{R}} \end{array}$$

**Figure 6:**
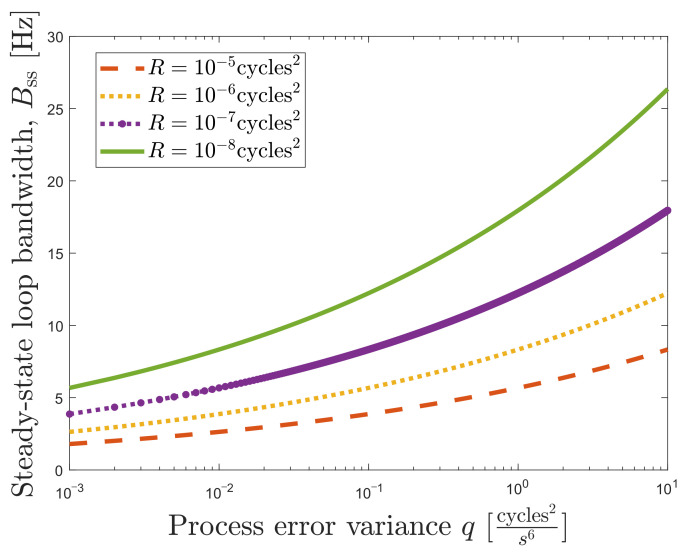
Relation between steady-state loop bandwidth $B_{\mathrm{ss}}$ and process error variance *q* in a third-order DSKF. ©IEEE. Adapted, with permission, from [20].

Figure 6 shows the relation between *q* and *B* for different values of *R* based on Equation (37). This relationship eases the LBCA implementation in the DSKF since the loop bandwidth update can be related with the covariances.

Finally, the relation between the $B_{\mathrm{ss}}$ and $K_{\mathrm{ss}}$ is calculated based on Equations (34) and (37):

(38) $$\begin{array}{c} K_{\mathrm{ss}}={\left[\begin{array}{ccc} 2\;\frac{6}{5}B_{\mathrm{ss}}\tau_{\mathrm{int}} & 2{\left(\frac{6}{5}B_{\mathrm{ss}}\right)}^{2}\tau_{\mathrm{int}} & {\left(\frac{6}{5}B_{\mathrm{ss}}\right)}^{3}\tau_{\mathrm{int}} \end{array}\right]}^{T} \end{array}$$

### 2.6. LUT-DSKF

**Figure 7:**
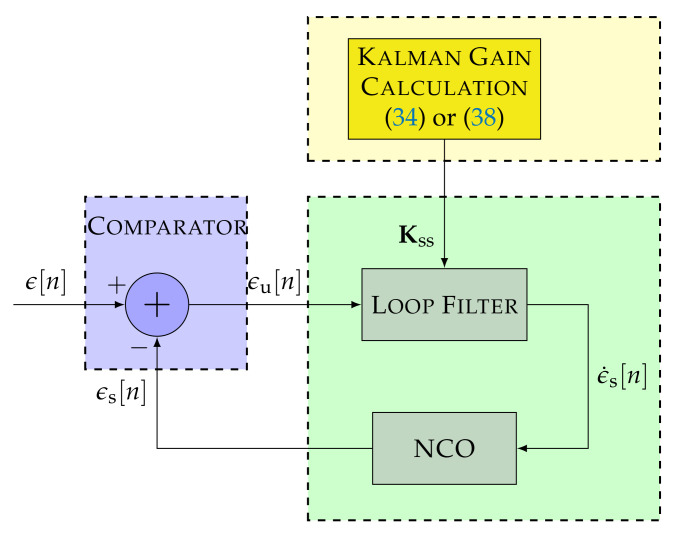
Linear model of lookup table (LUT)-DSKF.

Figure 7 shows the linear model of the LUT-DSKF. The LUT-DSKF is a simplified version of the DSKF that considers the steady-state convergence of K. The loop bandwidth *B* can be used to set the values of K using Equation (38). Alternatively, *q* and *R* can be selected to define K based on Equation (34).

## 3. Adaptive Techniques in DSKF

This section describes the three adaptive tracking techniques under evaluation: the *C*/*N*_0_-based DSKF, the LBCA-based DSKF, and the LBCA-based LUT-DSKF.

### 3.1. $C/N_{0}$-Based DSKF

**Figure 8:**
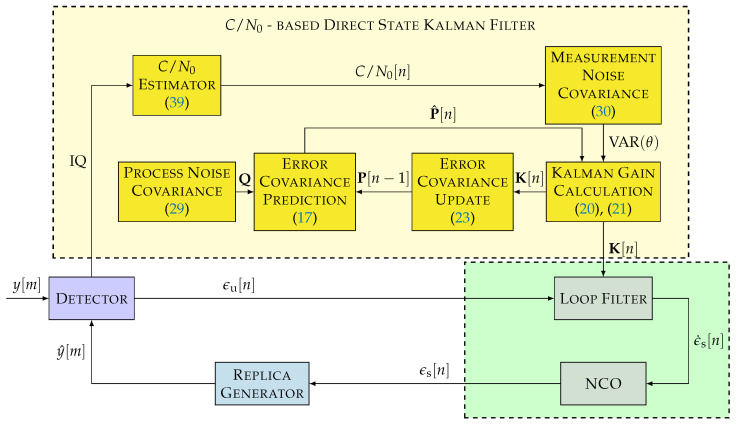
Non-linear model of the carrier-to-noise density ratio ($C/N_{0}$)-based DSKF.

Figure 8 shows the non-linear model diagram of the *C*/*N*_0_-based DSKF. This technique adapts the measurement noise covariance matrix R while fixing the process covariance matrix Q. In the case of a Costas PLL, the measurement noise covariance *R* depends on the *C*/*N*_0_ and the integration time $\tau_{\mathrm{int}}$ (see Equation (30)).

There are several methods to calculate the *C*/*N*_0_. The Beaulieu’s method is used due to its estimation accuracy and simplicity to estimate the *C*/*N*_0_ [41,42]. This method is sub-optimal if the carrier phase lock is not achieved. However, it is assumed that the carrier phase is in lock.

(39) $$\begin{array}{c} C/N_{0}=\frac{1}{\tau_{\mathrm{int}}}{\left(\frac{1}{N}\sum_{v=1}^{N}\frac{P_{n,v}}{P_{d,v}}\right)}^{-1} \end{array}$$

where *N* is the number of observed correlation samples, $P_{n,v}$ is the estimated noise power in the correlation, and $P_{d,v}$ is the signal-plus-noise power. $P_{n,v}$ is represented as:

(40) $$\begin{array}{c} P_{n,v}={\left(|I_{p}\left[v\right]|-|I_{p}\left[v-1\right]|\right)}^{2} \end{array}$$

$I_{p}$ is the prompt correlation value of the in-phase component. $P_{d,v}$ is defined as:

(41) $$\begin{array}{c} P_{d,v}=\frac{1}{2}\left(I_{p}{\left[v\right]}^{2}+I_{p}{\left[v-1\right]}^{2}\right) \end{array}$$

If the noise contribution is small, the instantaneous signal-plus-noise power $P_{d,v}$ approximates to the signal’s power.

### 3.2. LBCA-Based DSKF

**Figure 9:**
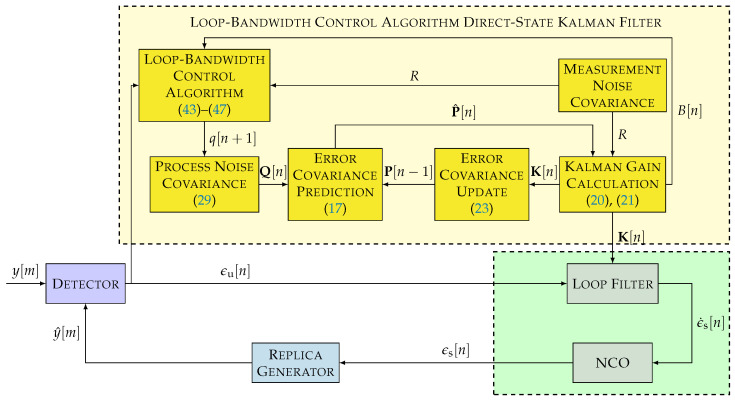
Non-linear model of loop-bandwidth control algorithm (LBCA)-based DSKF. ©IEEE. Adapted, with permission, from [20].

Figure 9 shows the architecture of the adaptive DSKF using the LBCA. This technique adapts the loop bandwidth *B* of the DSKF and, in turn, adapts *q* while setting *R* to a constant value based on Equation (37).

In previous studies, the LBCA has been implemented in the standard STL [28]. The LBCA-based STL presented superior tracking and system performance compared to other state-of-the-art techniques while achieving the lowest complexity [7]. This technique adapts the loop bandwidth based on a normalized bandwidth-dependent weighted difference between estimated noise and estimated signal dynamics. The normalized bandwidth $B_{N}$ is the product between the integration time $\tau_{\mathrm{int}}$ and the loop bandwidth *B*:

(42) $$B_{N}=B\;\tau_{\mathrm{int}}$$

First, the absolute mean $|\mu_{\epsilon_{u}}|$ and the standard deviation $\sigma_{\epsilon_{u}}$ of the discriminator’s output are estimated. Second, the normalized dynamics $\bar{D}$ are calculated:

(43) $$\bar{D}\left[n\right]=\frac{|\mu_{\epsilon_{u}}\left[n\right]|}{|\mu_{\epsilon_{u}}\left[n\right]|+\sigma_{\epsilon_{u}}\left[n\right]}$$

Third, the difference between the normalized dynamics and a normalized-bandwidth-dependent weighting function $g[n,B_{N}]$ is performed:

(44) $$c\left[n\right]=g_{\mathrm{Max}}\cdot \bar{D}\left[n\right]-g\left[n,B_{N}\right]$$

where *c* is the control value and $g_{\mathrm{Max}}$ indicates the maximum value of $g[n,B_{N}]$. Finally, the control value updates the current normalized bandwidth $B_{N}$:

(45) $$\hat{B}\left[n\right]=\frac{B_{N}\left[n\right]+c\left[n\right]}{\tau_{\mathrm{int}}}$$

where $\hat{B}$ is the updated loop bandwidth. The noisy mean and standard deviation estimates can induce some noise instabilities into the updated loop bandwidth. Therefore, a Schmitt trigger is included to reduce these noise instabilities. The Schmitt trigger changes the next loop bandwidth B[n+1] by $\Delta_{B}$ if the absolute difference between the updated loop bandwidth $\hat{B}\left[n\right]$ and the actual B[n] exceed $\Delta_{B}$:

(46) $$B\left[n+1\right]=\left\{\begin{array}{cc} 0 & \mathrm{if}\;n=0\\ \hat{B}\left[n\right]+\Delta_{B} & \mathrm{if}\;\hat{B}\left[n\right]-B\left[n\right]\geq \Delta_{B}\\ \hat{B}\left[n\right]-\Delta_{B} & \mathrm{if}\;B\left[n\right]-\hat{B}\left[n\right]\leq \Delta_{B}\;\\ B[n] & \mathrm{otherwise} \end{array}\right.$$

Based on the relationship between *R*, *q*, and the steady-state loop bandwidth $B_{\mathrm{ss}}$ from Equation (37), the updated *q* can be calculated:

(47) $$\begin{array}{c} q\left[n+1\right]=2.986\;B^{6}\left[n+1\right]\;R \end{array}$$

### 3.3. LBCA-Based LUT-DSKF

**Figure 10:**
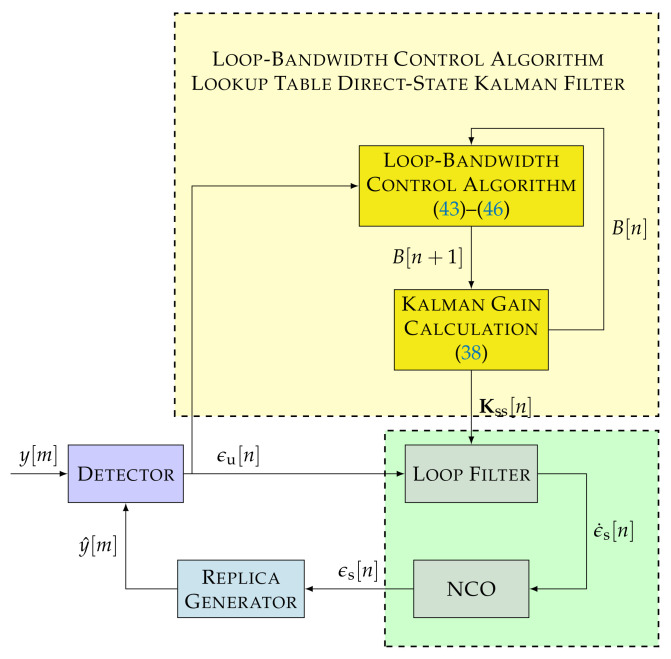
Non-linear model of LBCA-based LUT-DSKF.

The convergence of the DSKF’s Kalman gains at the steady-state region can reduce the complexity of the algorithm. From the DARE equation (see Equation (31)), the relation between the steady-state Kalman gains $K_{\mathrm{ss}}$, *q*, and *R* can be derived (see Equation (34)). This relation simplifies the algorithm as shown in Figure 7. Figure 10 presents the LBCA-based LUT-DSKF. The same steps as in the LBCA-based DSKF to calculate the updated loop bandwidth B[n+1] are followed (see Equations (43)–(46)). In contrast to the LBCA-based DSKF, the mapping between Kalman gains and loop bandwidth is directly done using Equation (38).

## 4. Experimental Setup

This section describes the GNSS receiver under test, the configuration of each presented adaptive algorithm, the metric used to determine the tracking and system performance, and the simulated scenarios.

### 4.1. Receiver and Algorithm Implementation

**Figure 11:**
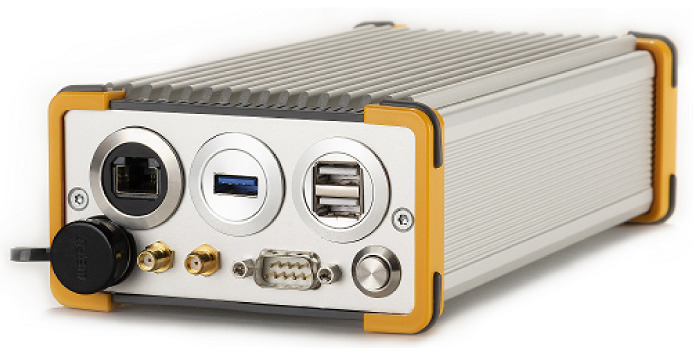
Photo of the GOOSE receiver @Fraunhofer IIS/Paul Pulkert.

The GOOSE© platform, developed by Fraunhofer IIS and marketed through TeleOrbit GmbH, is a GNSS receiver with an open software interface [29,43]. A picture of the receiver is shown in Figure 11. The receiver contains a customized tri-band radio-frequency front-end (RFFE), a Xilinx Kintex7 field-programmable gate array (FPGA), and a dualcore ARM processor. The RFFE amplifies, filters, downconverts, discretizes the GNSS signals, and sends the digital samples to the FPGA. The analog-to-digital converter (ADC) discretizes each frequency band at a sample rate of 81 MHz and a resolution of 8 bits. The FPGA includes one acquisition module and sixty tracking channels, which the processor can control. The processor performs the acquisition of the incoming digital samples using the acquisition module of the FPGA. The tracking starts once the acquisition achieves a rough estimate of the frequency Doppler $f_{d}$ and code phase τ. The tracking stage of this GNSS receiver is partially implemented in the FPGA (e.g., correlators and NCO) and software (e.g., discriminators and loop filters). This stage consists of three steps. First, the FLL and the DLL refine the acquired $f_{d}$ and τ estimates. Second, the PLL starts and synchronizes with the carrier phase. Finally, the FLL stops and the PLL works unaided when the latter successfully achieves a good lock with the carrier phase. The receiver synchronizes with the navigation data at this stage, and the integration time increases to the symbol period. In the case of Global Positioning System (GPS) L1 C/A, the integration time is increased to 20 ms.

The *C*/*N*_0_-based DSKF, the LBCA-based DSKF, and the LBCA-based LUT-DSKF are implemented in the third-order Costas PLL of the GOOSE receiver in software.

#### 4.1.1. $C/N_{0}$-Based DSKF Configuration

The time of response of the *C*/*N*_0_ estimation determines the agility of the measurement’s covariance update. This parameter changes according to the accumulated correlation samples *N* (see Equation (39)). This study evaluates this method’s tracking and system performance with N=100 samples and N=500 samples. Using an integration time $\tau_{\mathrm{int}}$ of 20 ms, the response time of the *C*/*N*_0_’s estimate is 2 s and 10 s, respectively. The steady-state process variance *q* is set to a constant value. Different values of *q* are evaluated since this parameter directly impacts on the robustness to signal dynamics.

#### 4.1.2. LBCA-Based DSKF and LUT-DSKF Configuration

**Figure 12:**
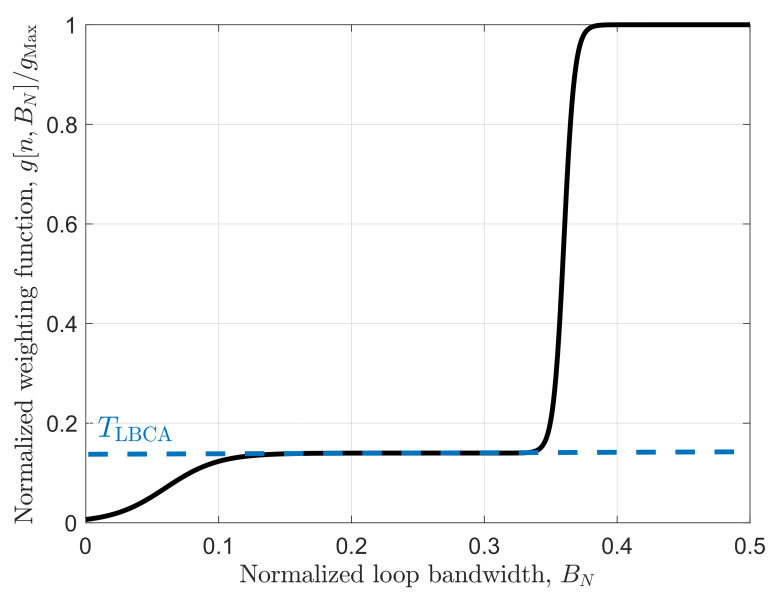
Selected Normalized weighting function in LBCA-based techniques.

The selected weighting function $g[n,B_{N}]$ is a linear combination of two Sigmoid functions and has the following expression:

(48) $$g\left[n,B_{N}\right]={\left[\begin{array}{c} 0.014\\ 0.086 \end{array}\right]}^{T}\;\left[\begin{array}{c} \mathrm{Sig}\left(50\left(B_{N}-0.06\right)\right)\\ \mathrm{Sig}\left(250\left(B_{N}-0.36\right)\right) \end{array}\right]$$

where Sig(·) is the Sigmoid function [44]. Figure 12 shows the graphical representation of Equation (48). $T_{\mathrm{LBCA}}$ is the normalized dynamic threshold and indicates the sensitivity of the algorithm to signal dynamics. In this case, $g_{\mathrm{Max}}$ is set to 0.1 and $T_{\mathrm{LBCA}}$ to 0.14. This weighting function presented the best results in the LBCA-based PLL [7].

In the LBCA-based DSKF, *R* is set to 10${}^{-7}$ cycles${}^{2}$. From Figure 6 and Figure 12, one can observe that *q* varies between 10${}^{-3}$ cycles${}^{2}/$s${}^{6}$ and 10 cycles${}^{2}/$s${}^{6}$, considering $\tau_{\mathrm{int}}=\;$20 ms.

### 4.2. Performance Metric

The metric to evaluate the PLL tracking and system performance is the same as in previous studies [7]. The tracking performance $P_{\mathrm{Tracking}}$ evaluates the tracking of a single satellite vehicle ( SV), whereas the system performance $P_{\mathrm{System}}$ considers all the visible SV. $P_{\mathrm{Tracking}}$ in meters is characterized as:

(49) $$P_{\mathrm{Tracking}}=\left({\bar{\sigma }}_{\theta }^{u}-\sigma_{\mathrm{LB}}^{u}\right)\cdot \lambda_{L1}$$

where $\lambda_{L1}$ is the wavelength of the GPS L1 C/A signal, ${\bar{\sigma }}_{\theta }^{u}$ is the average of the last ten minutes un-smoothed carrier phase error’s standard deviation, and $\sigma_{\mathrm{LB}}^{u}$ is the square root of the CRB [7]. ${\bar{\sigma }}_{\theta }^{u}$ in cycles is defined as:

(50) $${\bar{\sigma }}_{\theta }^{u}=\frac{1}{K_{\mathrm{sim}}}\sum_{k=1}^{K_{\mathrm{sim}}}\sigma_{\theta }^{u}\left[k\right]$$

where $K_{\mathrm{sim}}$ is the number of evaluation epochs in samples. The total evaluation time $T_{\mathrm{sim}}$ in seconds is:

(51) $$T_{\mathrm{sim}}=\frac{K_{\mathrm{sim}}}{f_{\log}}$$

where $f_{\log}$ is the data-logging rate in Hertz.

$\sigma_{\mathrm{LB}}^{u}$ in cycles is represented as:

(52) $$\sigma_{\mathrm{LB}}^{u}=\left(\frac{1}{2\pi }\right)\sqrt{\frac{1}{2\tau_{\mathrm{int}}C/N_{0}}\left(1+\frac{1}{2\tau_{\mathrm{int}}C/N_{0}}\right)}$$

where the $\left(1+\frac{1}{2\tau_{\mathrm{int}}C/N_{0}}\right)$ term is the squaring loss [1].

The three-sigma rule-of-thumb conservative upper threshold $\sigma_{\theta^{u}}^{th}$ is considered in the tracking performance evaluation. For a two-quadrant phase discriminator, $\sigma_{\theta^{u}}^{th}$ in cycles is expressed as:

(53) $$\sigma_{\theta^{u}}^{th}=\frac{1}{24}$$

A low value of $P_{\mathrm{Tracking}}$ denotes good tracking performance. If the measured $P_{\mathrm{Tracking}}$ from ${\bar{\sigma }}_{\theta }^{u}$ is less than $P_{\mathrm{Tracking}}$ considering $\sigma_{\theta^{u}}^{th}$, one can ensure stable tracking and no cycle slips [1]. On the contrary, if the measured $P_{\mathrm{Tracking}}$ is bigger than the conservative threshold, the probability of losing the lock increases.

The metric used for the system performance $P_{\mathrm{System}}$ is expressed as:

(54) $$\begin{array}{c} P_{\mathrm{System}}=\bar{\mathrm{PLI}}\times {\bar{N}}_{\mathrm{sat}} \end{array}$$

where $\bar{\mathrm{PLI}}$ is the average of the phase-lock indicator (PLI) with respect $K_{\mathrm{sim}}$ and the accumulated number of tracked SV $N_{\mathrm{sat}}^{\mathrm{Acc}}$, and ${\bar{N}}_{\mathrm{sat}}$ is the normalized average number of visible satellites being tracked.

The expression of $\bar{\mathrm{PLI}}$ is:

(55) $$\bar{\mathrm{PLI}}=\frac{1}{K_{\mathrm{sim}}\;N_{\mathrm{sat}}^{\mathrm{Acc}}}\sum_{k=1}^{K_{\mathrm{sim}}}\sum_{l=1}^{N_{\mathrm{Sat}}^{\mathrm{Acc}}}{\mathrm{PLI}}^{l}\left[k\right]$$

and ${\mathrm{PLI}}^{l}\left[n\right]$ is calculated based on the prompt in-phase $I_{p}^{l}$ and prompt quadrature $Q_{p}^{l}$ correlation samples of the corresponding $l^{th}$ SV:

(56) $${\mathrm{PLI}}^{l}=\frac{{\left(I_{p}^{l}\right)}^{2}-{\left(Q_{p}^{l}\right)}^{2}}{{\left(I_{p}^{l}\right)}^{2}+{\left(Q_{p}^{l}\right)}^{2}}$$

The second term of Equation (54), ${\bar{N}}_{\mathrm{sat}}$, is defined as:

(57) $$\begin{array}{c} {\bar{N}}_{\mathrm{sat}}=\frac{1}{K_{\mathrm{sim}}N_{\mathrm{sat}}^{\mathrm{total}}}\sum_{k=1}^{K_{\mathrm{sim}}}N_{\mathrm{sat}}\left[k\right] \end{array}$$

where $N_{\mathrm{sat}}^{\mathrm{total}}$ is the total number of SV during the simulation.

$\bar{\mathrm{PLI}}$ and ${\bar{N}}_{\mathrm{sat}}$ are normalized. Consequently, $P_{\mathrm{System}}$ is also normalized. A value near to one of $P_{\mathrm{System}}$ refers to good system performance, whereas a value near zero indicates poor system performance and the possibility of not achieving a PVT solution. The average of $P_{\mathrm{System}}$ with respect to the *C*/*N*_0_ levels leads to a comprised metric ${\bar{P}}_{\mathrm{System}}$ that evaluates the overall system performance of an adaptive tracking technique:

(58) $$\begin{array}{c} {\bar{P}}_{\mathrm{System}}=\frac{1}{N_{C/N_{0}}}\sum_{n=1}^{N_{C/N_{0}}}P_{\mathrm{System}}^{n} \end{array}$$

where $N_{C/N_{0}}$ is the number of *C*/*N*_0_ levels.

### 4.3. Evaluation Setup

The evaluation setup is the same as in previous studies [7,20,28]. The Spirent GSS9000 radio-frequency constellation simulator (RFCS) generates controlled scenarios at different *C*/*N*_0_ and signal dynamics levels. The simulator is configured to perform 20 min simulations of a specific scenario at eight different *C*/*N*_0_ levels ($N_{C/N_{0}}=8$). In contrast to previous studies, the selected *C*/*N*_0_ levels are {25, 29, 33, 37, 41, 45, 48, 52} dBHz. The simulation always starts at the highest *C*/*N*_0_ level of 52 dBHz to ensure successful signal acquisition. Next, the *C*/*N*_0_ level is reduced in 30 second intervals until reaching the desired *C*/*N*_0_ level. The tracking and system performance are measured considering the last ten minutes of the simulation, $T_{\mathrm{sim}}=$ 600 s, in which the correct *C*/*N*_0_ level is assured.

**Figure 13:**
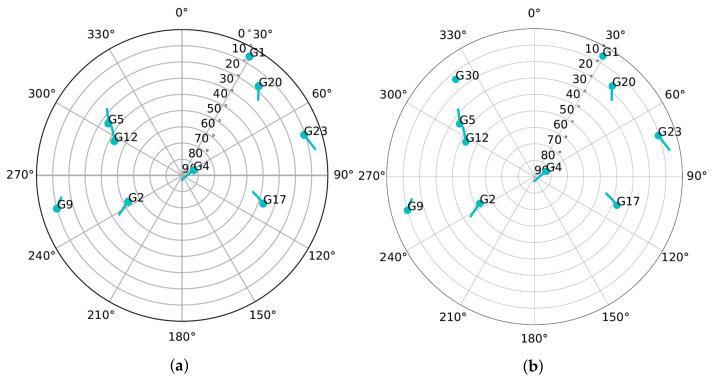
Sky-plot of simulated scenario. (**a**) Static Scenario. (**b**) Dynamic Scenario.

A static scenario and a dynamic scenario are selected to evaluate the adaptive tracking techniques. The static scenario represents stationary use-cases such as GNSS reference stations, and the dynamic scenario presents harsh vehicular conditions. Figure 13 shows the sky-plots of both scenarios. In the static scenario there are nine SVs during the entire simulation (see Figure 13a). However, SV G1 disappears behind the horizon after two minutes of the simulation. The dynamic scenario shows ten visible SVs (see Figure 13b). As in the static scenario, SV G1 disappears after two minutes of the simulation. Moreover, SV G30 rises above the horizon near the end of the simulation. Therefore, to avoid these transient SVs, SV G1 and SV G30 are not considered, and $N_{\mathrm{sat}}^{\mathrm{total}}$ is set to eight for both scenarios.

The same SV as previous research are selected to evaluate the tracking performance [7]. The GPS L1 C/A signal of SV G4 is selected to evaluate the static tracking performance, and the GPS L1 C/A signal of SV G17 is assigned for the dynamic tracking performance. The maximum line-of-sight (LOS) signal dynamics for the dynamic simulated scenario is 8.7 g/s [7].

## 5. Results

This section evaluates the tracking performance, system performance, number of operations, and time complexity of the adaptive tracking techniques in the DSKF. The dataset used to plot the presented results are available on the cloud [45].

### 5.1. Static Scenario

**Figure 14:**
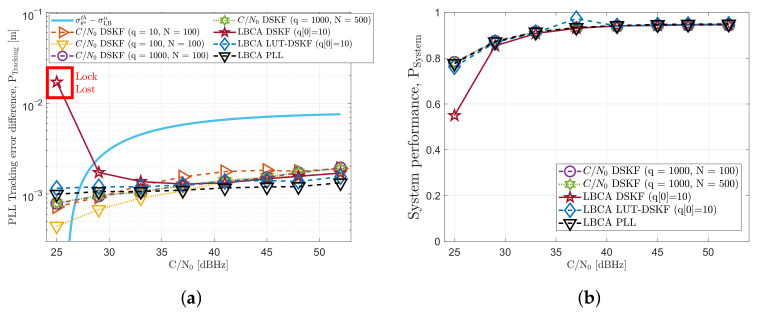
Static performance of adaptive tracking techniques in third-order Costas phase locked loop (PLL) at different *C*/*N*_0_ levels. (**a**) Tracking performance of satellite vehicle (SV) G4. (**b**) System performance.

Figure 14 presents the tracking performance $P_{\mathrm{Tracking}}$ and system performance $P_{\mathrm{System}}$ of the implemented adaptive techniques in a stationary scenario. Four configurations of the *C*/*N*_0_-based DSKF using different steady-state process noise variances (q={10,100,1000}) and different correlation samples (N={100,500}) are evaluated. Moreover, the LBCA-based DSKF, and the LBCA-based LUT-DSKF are analyzed using the weighting function defined in Equation (48). These adaptive tracking techniques are compared with the LBCA-based STL using the same weighting function.

Figure 14a shows that all the presented configurations of the *C*/*N*_0_-based DSKF do not lose the lock at any *C*/*N*_0_ level. The number of correlation samples *N* does not impact the static tracking performance. In contrast, *q* has an effect at low *C*/*N*_0_ levels. An intermediate value of q=100${\mathrm{cycles}}^{2}/s^{6}$ achieves the lowest results, meaning the best tracking performance. In the LBCA-based DSKF, *q* starts with an initial value of 10 ${\mathrm{cycles}}^{2}/s^{6}$ and is adapted based on the LBCA’s loop bandwidth update, while *R* is fixed to $10^{-7}{\mathrm{cycles}}^{2}$. At high *C*/*N*_0_ levels, this method presents the same tracking performance as the other techniques. However, at low *C*/*N*_0_ levels, $P_{\mathrm{Tracking}}$ increases significantly, until finally losing the lock at 25 dBHz. The LBCA-based LUT-DSKF does not lose the lock at low *C*/*N*_0_ levels, but it does not perform better than the *C*/*N*_0_-based DSKF. The LBCA-based STL performs best at high *C*/*N*_0_ levels, but the performance deteriorates at low *C*/*N*_0_ levels. The weighting function of the LBCA is configured to be robust to signal dynamics, with the cost of a worse performance at low *C*/*N*_0_ levels.

Figure 14b discloses each adaptive tracking technique’s system performance (see Equation (54)). All the configurations show a good system performance above 0.8, except for the LBCA-based DSKF at 25 dBHz. While the system performance of this technique at 25 dBHz is lower than 0.6, the PVT has not been lost.

### 5.2. Dynamic Scenario

**Figure 15:**
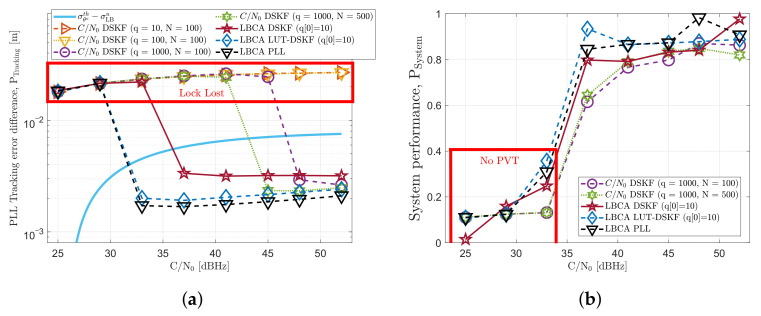
Dynamic performance of adaptive tracking techniques in third-order Costas PLL at different *C*/*N*_0_ levels. (**a**) Tracking performance of SV G17. (**b**) System performance.

Figure 15 displays the tracking and system performance of the adaptive techniques in a dynamic scenario. SV G17 is challenging to track due to the high LOS jerk dynamics. From Figure 15a, it is observed that a high *q* and *N* are required in the *C*/*N*_0_-based DSKF to maintain the carrier phase synchronization lock. The *C*/*N*_0_-based DSKF achieves best tracking performance with q=1000${\mathrm{cycles}}^{2}/s^{6}$ and a N= 500 samples, maintaining the lock until 45 dBHz. The LBCA-based DSKF keeps the lock until 38 dBHz. However, this technique presents an error bias compared to the other techniques. This bias exists due to the transitions between the update of *q* and the Kalman gains’ convergence. In contrast to the latter technique, the LBCA-based LUT-DSKF does not contain any error bias, and it performs almost as well as the LBCA-based PLL, keeping the lock until 33 dBHz. The LBCA-based PLL has a superior dynamic tracking performance than the other techniques due to the direct relation between loop bandwidth and filter coefficients. This direct relationship leads to a faster reaction of high signal dynamics.

The results of the dynamic system performance (see Figure 15b) are consonant with the dynamic tracking performance. All the adaptive tracking techniques can achieve a PVT solution at high *C*/*N*_0_ levels (38–52 dBHz). The LBCA-based LUT-DSKF and the LBCA-based PLL have the best system performance results, whereas the *C*/*N*_0_-based DSKF technique shows poor performance. At low *C*/*N*_0_ levels, the LBCA-based techniques almost achieve a continuous PVT at 33 dBHz. In contrast to the LBCA-based methods, the *C*/*N*_0_-based DSKF only achieves a continuous tracking of one SV.

### 5.3. Total System Performance

Table 1 summarizes the obtained results from Figure 14b and Figure 15b using the average system performance ${\bar{P}}_{\mathrm{System}}$ presented in Equation (58). The *labels* column of this table is used for further analysis in the discussion. Furthermore, the best static and dynamic system performance is marked green.

### 5.4. Complexity

Table 2 shows the added number of additions, multiplications, and divisions required for each adaptive technique in the DSKF compared to the standard STL. The total number of operations is marked as red, orange, and green, depending on the level of complexity. The colors vary from the most complex one, marked in red, to the simplest one, marked in green.

The presented equivalence between the DSKF and the standard STL (see Figure 4 and Figure 5) shows that only the Kalman gain calculation (i.e., yellow block of Figure 4) must be included. The *C*/*N*_0_-based DSKF adapts *R* based on Equation (30), being *q* a constant value. In contrast, the LBCA-based DSKF set *R* to a constant value, while adapting *q* using the LBCA. This technique needs additional complexity to map the loop bandwidth with the steady-state process variance *q* (see Equation (47)).

The LUT-DSKF significantly reduces the complexity by calculating the convergence of the Kalman gains K in the steady-state. In the LBCA-based LUT-DSKF, the relation between the *B* and K is used (see Equation (38)) to update the filter coefficients.

In all the LBCA-based techniques, the weighting function of the LBCA technique is approximated using the piecewise linear approximation of nonlinearities (PLAN) technique [7].

Table 3 shows the time complexity of the standard PLL and each adaptive tracking technique. The time complexity measures the algorithm’s processing time in software on the processing platform. The same procedure as in previous research is carried out [7]. An Intel Skylake micro-architecture with a clock speed of 3700 MHz is used to evaluate the time complexity of each technique. The code is implemented in C++, and a for-loop is used to iterate the operation of a loop filter $3\times 10^{8}$ times. The processing time of each method is measured using the *chrono* library [46], and the *operf* Linux profiler tool is used to analyze the utilization of the used libraries during the algorithm’s execution [47]. In addition, the *taskset* command is used to bind the application process to one single core [48].

Three parameters are shown in Table 3. First, the total time complexity $T_{C}$ in seconds. Second, the average time complexity at each iteration $T_{\mathrm{Iter}}$ in nanoseconds:

(59) $$\begin{array}{c} T_{\mathrm{Iter}}=\frac{T_{C}}{3\times 10^{8}}\times 10^{9}\;\left[\mathrm{ns}\right] \end{array}$$

Finally, the added time complexity $T_{\mathrm{Added}}$ compared to the standard loop filter $T_{C}^{\mathrm{Standard}}$:

(60) $$\begin{array}{c} T_{\mathrm{Added}}=\frac{T_{C}}{T_{C}^{\mathrm{Standard}}}\;\left[\mathrm{times}\right] \end{array}$$

While this approach depends on how exemplary the algorithm’s software implementation is, the results show a correlation between the number of operations and the time complexity.

## 6. Discussion

**Figure 16:**
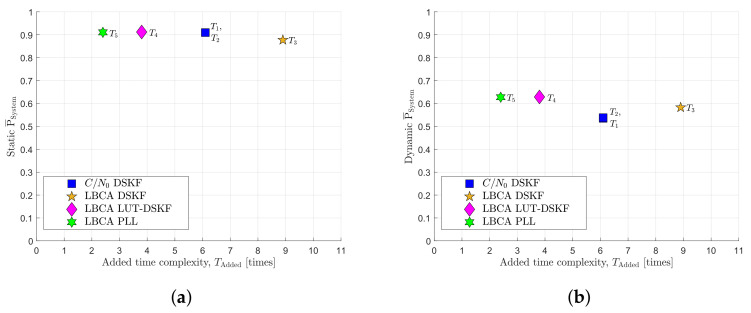
System performance vs. added time complexity comparison. (**a**) Static scenario. (**b**) Dynamic scenario.

Figure 16 compares the average system performance ${\bar{P}}_{\mathrm{System}}$ and the added time complexity $T_{\mathrm{Added}}$ of the presented adaptive tracking techniques implemented in a third-order DSKF for carrier phase tracking. These graphs summarize the collected results combining Table 1 and Table 3.

Figure 16a shows that the LBCA-based DSKF $T_{3}$ have the worst ${\bar{P}}_{\mathrm{System}}$ and the highest $T_{\mathrm{Added}}$. In contrast, the LBCA-based LUT-DSKF $T_{4}$ achieves the best static ${\bar{P}}_{\mathrm{System}}$, although its complexity is higher than the LBCA-based standard PLL $T_{5}$. The state-of-the-art *C*/*N*_0_-based DSKF $T_{1},T_{2}$ have similar ${\bar{P}}_{\mathrm{System}}$, and $T_{\mathrm{Added}}$ is between $T_{4}$ and $T_{3}$. $T_{5}$ presents the lowest $T_{\mathrm{Added}}$ with a slightly worse ${\bar{P}}_{\mathrm{System}}$ than $T_{4}$.

In the dynamic scenario, the superior performance of the LBCA-based techniques is clearly observed in Figure 16b. In this case, $T_{1}$ and $T_{2}$ present the worst ${\bar{P}}_{\mathrm{System}}$. In a highly dynamic event, the carrier tracking loop cannot follow the phase dynamics, leading to a fast shift from in-phase to quadrature component. In such a moment, the *C*/*N*_0_ estimate of Beaulieu’s method drops. Consequently, *R* increases, and the DSKF wrongly stops trusting the incoming carrier phase measurement. This leads to a loss of the carrier lock. In Figure 15a, a greater number of the accumulated correlation samples *N* and *q* improves the robustness to dynamics. Then, a further evaluation with higher *q* and *N* should improve ${\bar{P}}_{\mathrm{System}}$ in the dynamic scenario. Among the LBCA-based techniques, $T_{3}$ performs the worst. Once $T_{3}$ adapts *q*, time is needed to adjust the Kalman gains to the steady-state values. Due to this latency, $T_{3}$ is not fast enough to adapt its coefficients in time, leading to a higher risk of losing the lock at high dynamic scenarios. $T_{4}$ experiences no latencies since the steady-state Kalman gains are directly set based on the loop bandwidth update. This method achieves the best ${\bar{P}}_{\mathrm{System}}$, followed by $T_{5}$.

The scope of this research is to show the extension of the LBCA by applying it to the DSKF. While this has been shown, adding the LBCA into the DSKF to adapt *q* while fixing *R* does not present remarkable results, and the complexity increases significantly. Therefore, other methods have been considered to improve the performance and reduce the complexity. The LBCA-based LUT-DSKF presents superior static and dynamic ${\bar{P}}_{\mathrm{System}}$ among the rest of the adaptive tracking techniques. However, the LBCA-based PLL is less complex than the LBCA-based LUT DSKF, with a slight reduction of the total system performance in both static and dynamic scenarios.

## 7. Conclusions

This study presents the first proof of the LBCA’s general applicability to any control system by implementing it in the DSKF. First, the relation between the standard STL and the DSKF is presented. Second, DARE’s solution relates the DSKF’s loop bandwidth, the noise covariances, and the Kalman gains in the steady-state. This relationship eases the implementation of the LBCA into the DSKF, and two approaches are presented: The LBCA-based DSKF and the LBCA-based LUT-DSKF. The LBCA-based DSKF uses the LBCA to adapt *q* for each loop iteration while setting *R* to a constant value. In contrast, the LBCA-based LUT-DSKF directly relates the LBCA’s loop bandwidth update and the Kalman gains. These adaptive tracking techniques have been evaluated in a third-order Costas PLL. These techniques’ complexity and static and dynamic system performance are compared with the state-of-the-art *C*/*N*_0_-based DSKF and the LBCA-based standard STL [7]. Results show the superior performance of the LBCA-based LUT-DSKF compared to the other techniques. In terms of complexity, the LBCA-based LUT-DSKF presents less complexity compared to the other adaptive DSKFs. However, this method has greater added time complexity than the LBCA-based standard PLL. In further research, Equation (38) will be merged into the STL to achieve similar time complexity.

An extension of the presented tracking schemes is the implementation of a LUT-DSKF that encloses the DLL, FLL, and PLL of a tracking channel. The use of the LBCA in this tracking architecture can improve the tracking performance in scenarios with different noise and signal dynamics. To cope with scenarios with multipath effects (e.g., in urban scenarios), a multi-frequency LUT-DSKF will be proposed, and the addition of the LBCA will be evaluated. Furthermore, an evaluation in real scenarios of all the LBCA-based techniques will lead to the final verification of the LBCA.

This paper opens the door to a range of applications of the LBCA where the KF is involved. The LBCA can be implemented in the tracking stage, the interference mitigation stage (e.g., adaptive notch filtering), and the post-processing stage (e.g., adaptive loose and tightly coupling solutions) of a GNSS receiver.

## Figures and Tables

**Table 1:** Overall system performance of adaptive tracking techniques.

Tracking	Configuration	Label	${\bar{P}}_{\mathrm{System}}$	${\bar{P}}_{\mathrm{System}}$
Technique			Static	Dynamic
$C/N_{0}$-based DSKF	q=1000, N=100	$T_{1}$	0.910	0.535
	q=1000, N=500	$T_{2}$	0.909	0.538
LBCA-based DSKF	$g_{\mathrm{Max}}=0.1$ , $T_{\mathrm{LBCA}}=0.14$	$T_{3}$	0.877	0.583
LBCA-based LUT-DSKF	$g_{\mathrm{Max}}=0.1$ , $T_{\mathrm{LBCA}}=0.14$	$T_{4}$	0.912	0.628
LBCA-based PLL	$g_{\mathrm{Max}}=0.1$ , $T_{\mathrm{LBCA}}=0.14$	$T_{5}$	0.911	0.627

**Table 2:** Complexity of adaptive tracking techniques based on the added number of operations.

Tracking	Sub-	Added Number of Operations:		
Technique	Module	Additions	Multiplications	Divisions
$C/N_{0}$-based DSKF	Error Cov. Prediction (17)	27	18	-
	Kalman Gain Calculation (20) and (21)	1	3	1
	Error Cov. Update (23)	9	9	-
	Measurement Noise Cov. (30)	1	5	2
	**Total**	38	35	3
LBCA-based DSKF	Error Cov. Prediction (17)	27	18	-
	Kalman Gain Calculation (20) and (21)	1	3	1
	Error Cov. Update (23)	9	9	-
	LBCA + PLAN [7]	6	7	1
	*q* and *B* relation (47)	0	6	0
	**Total**	45	37	2
LBCA-based LUT-DSKF	LBCA + PLAN [7]	6	7	1
	K and *B* relation (38)	0	9	0
	**Total**	6	16	1
LBCA-based PLL	LBCA + PLAN [7]	6	7	1
	**Total**	6	7	1

**Table 3:** Time complexity of adaptive tracking techniques, $3\times 10^{8}$ iterations.

Tracking	Total Time Complexity	Iteration Time Complexity	Added Time Complexity
Technique	$T_{C}$ [s]	$T_{\mathrm{Iter}}$ [ns]	$T_{\mathrm{Added}}$ [times]
Standard PLL	6.8	22.7	1
$C/N_{0}$-based DSKF	41.5	138.3	6.1×
LBCA-based DSKF	61.1	203.7	8.9×
LBCA-based LUT-DSKF	25.8	86	3.8×
LBCA-based PLL [7]	16.1	53.7	2.4×

## Data Availability

Publicly available datasets were analyzed in this study. This data can be found here: https://owncloud.fraunhofer.de/index.php/s/LGoWPVtV5xbQ9mB (accessed on 14 December 2020).

## Abbreviations

The following abbreviations are used in this manuscript:

ADC	analog-to-digital converter
BET	backward Euler transform
$C/N_{0}$	carrier-to-noise density ratio
CRB	Cramér-Rao bound
DARE	discrete algebraic Riccati equation
DLL	delay locked loop
DSKF	direct-state Kalman filter
ESKF	error-state Kalman-filter
FAB	fast adaptive bandwidth
FLL	frequency locked loop
FPGA	field-programmable gate array
GNSS	global navigation satellite system
GPS	Global Positioning System
IIR	infinite impulse response
KF	Kalman filter
LBCA	loop-bandwidth control algorithm
LOS	line-of-sight
LUT	lookup table
MMSE	minimum mean square error
NCO	numerically controlled oscillator
PLAN	piecewise linear approximation of nonlinearities
PLI	phase-lock indicator
PLL	phase locked loop
PVT	position, velocity, and time
RFCS	radio-frequency constellation simulator
RFFE	radio-frequency front-end
SSM	state space model
STL	scalar tracking loop
SV	satellite vehicle
